# Agnostic polygenic prediction of weight loss after bariatric surgery

**DOI:** 10.1172/jci.insight.198133

**Published:** 2026-03-19

**Authors:** Bastien Vallée Marcotte, Juan de Toro-Martín, André Tchernof, Louis Pérusse, Simon Marceau, Marie-Claude Vohl

**Affiliations:** 1 Centre Nutrition, santé et société (NUTRISS) - Institut sur la nutrition et les aliments fonctionnels (INAF),; 2 School of Nutrition,; 3 Quebec Heart and Lung Institute Research Centre,; 4 Department of Kinesiology, Faculty of Medicine, and; 5 Department of Surgery, Faculty of Medicine, Université Laval, Quebec City, Quebec, Canada.

**Keywords:** Gastroenterology, Genetics, Metabolism, Genetic risk factors, Obesity, Surgery

## Abstract

A large interindividual variability in weight loss outcomes following bariatric surgery is reported. To ensure optimal management of patients, it is crucial to accurately identify candidates most likely to benefit the most from the intervention. Since genetic variants largely contribute to surgery response, polygenic scores (PGS) derived from genome-wide association studies (GWAS) could constitute valuable tools for clinical decision making. We developed and evaluated PGS to predict the weight loss response in 540 patients with a body mass index (BMI) of 35 kg/m^2^ or higher who underwent biliopancreatic diversion with duodenal switch. Summary statistics derived from BMI-derived GWAS, together with summary statistics from previously published GWAS of BMI and adiposity features, were used to construct, evaluate, and benchmark weight loss PGS. The full-adjusted BMI PGS model built in the entire cohort explained 39.6% of the mean-over-time excessive body weight loss (%EBWL), while the BMI-PGS built in the training dataset explained 38.9%. All benchmarked PGS based on BMI showed a significant relationship with mean-over-time %EBWL. These findings highlight the potential of BMI PGS in predicting weight loss after bariatric surgery and support their use as promising tools to improve the effectiveness of future antiobesity treatments.

## Abstract 2

Polygenic risk scores can constitute effective tools to predict the weight loss response following bariatric surgery

## Introduction

Bariatric surgery has been proven to be an effective intervention to improve the quality of life and longevity in patients with refractory obesity (1, 2). Despite presenting numerous benefits that extend beyond weight loss, it is also associated with some risks (3, 4). The procedure is nonreversible and associated with long-term adverse effects on health, such as nutritional deficiency, dumping syndrome, and gastrointestinal discomfort (5). Patients who undergo the intervention usually need life-long vitamin supplementation in conjunction with a multidisciplinary medical follow-up (5).

Consequently, biliopancreatic diversion may not be the best solution for every eligible patient; hence, it is important to correctly identify patients who would be eligible for more conservative treatment options, including other types of surgeries or pharmacotherapy. Contraindications generally taken into account include low initial body mass index (BMI), high risk of nonobservance to vitamin supplementation, irritable bowel disease, ongoing major mental health issue, eating disorder, substance abuse, and coagulopathy (6). However, another important element to consider in eligibility assessment is the individual potential for weight loss. A large interindividual variability observed in weight loss is observed among patients in the years following the surgery. Such heterogeneity is partly influenced by surgical modality, sex, ethnicity, initial BMI, age, and genetic factors (4, 7–9).

To investigate the genetic contribution to weight loss variability, our research group previously constructed a polygenic risk score (PGS) of the percentage excess body weight loss (%EBWL) using single-nucleotide polymorphisms (SNPs) previously associated with BMI (8). To do so, 865 patients who underwent biliopancreatic diversion with duodenal switch had their body weight systematically tracked over the course of 48 months and were genotyped for 186 selected SNPs, identified as significantly associated with BMI in a previous genome-wide association study (GWAS) (10). A logistic prediction model for %EBWL comprising initial BMI, age, sex, and surgical modality area under the receiver operating characteristics (ROC) curve adjusted for optimism ([ΔAUC] = 0.867) significantly improved with the inclusion of the PGS (increase in ΔAUC = 0.021; 95% CI = 0.005–0.038) (8). The addition of the PGS to the model also enhanced the cost-effectiveness of bariatric surgery by lowering the false negative rate from 20.4% to 10.9% (8).

Notwithstanding these promising observations, a different approach was followed in the present study. Unlike our previous candidate-like study (10), where specific BMI-associated SNPs were used then to construct and test PGS models, we herein constructed and tested different PGS based on genome-wide results using a chip containing around 1.8 million genetic markers in order to attain exhaustive genome coverage. Our primary objective was to evaluate the ability of PGS derived from BMI GWAS to predict weight loss response following bariatric surgery. In addition, we assessed and benchmark the predictive performance of PGS derived from previously BMI and adiposity GWAS.

## Results

### Subject characteristics.

The study population before participant exclusion consisted of 565 participants (Supplemental Table 1; supplemental material available online with this article; https://doi.org/10.1172/jci.insight.198133DS1). Baseline characteristics of the 540 participants finally included in the analyses are presented in Table 1. The study population consisted of 150 men and 390 women. The majority of participants (96.1%) had class 3 obesity, with a mean BMI of 50.2 ± 7.1. Follow-up data on %EBWL were available for the majority of participants across multiple postoperative time points, with high data completeness over the 7-year follow-up period. Most of the participants had 5 or more follow-up time points (*n* = 495; 91.7%).

### Weight loss response.

**Figure 1:**
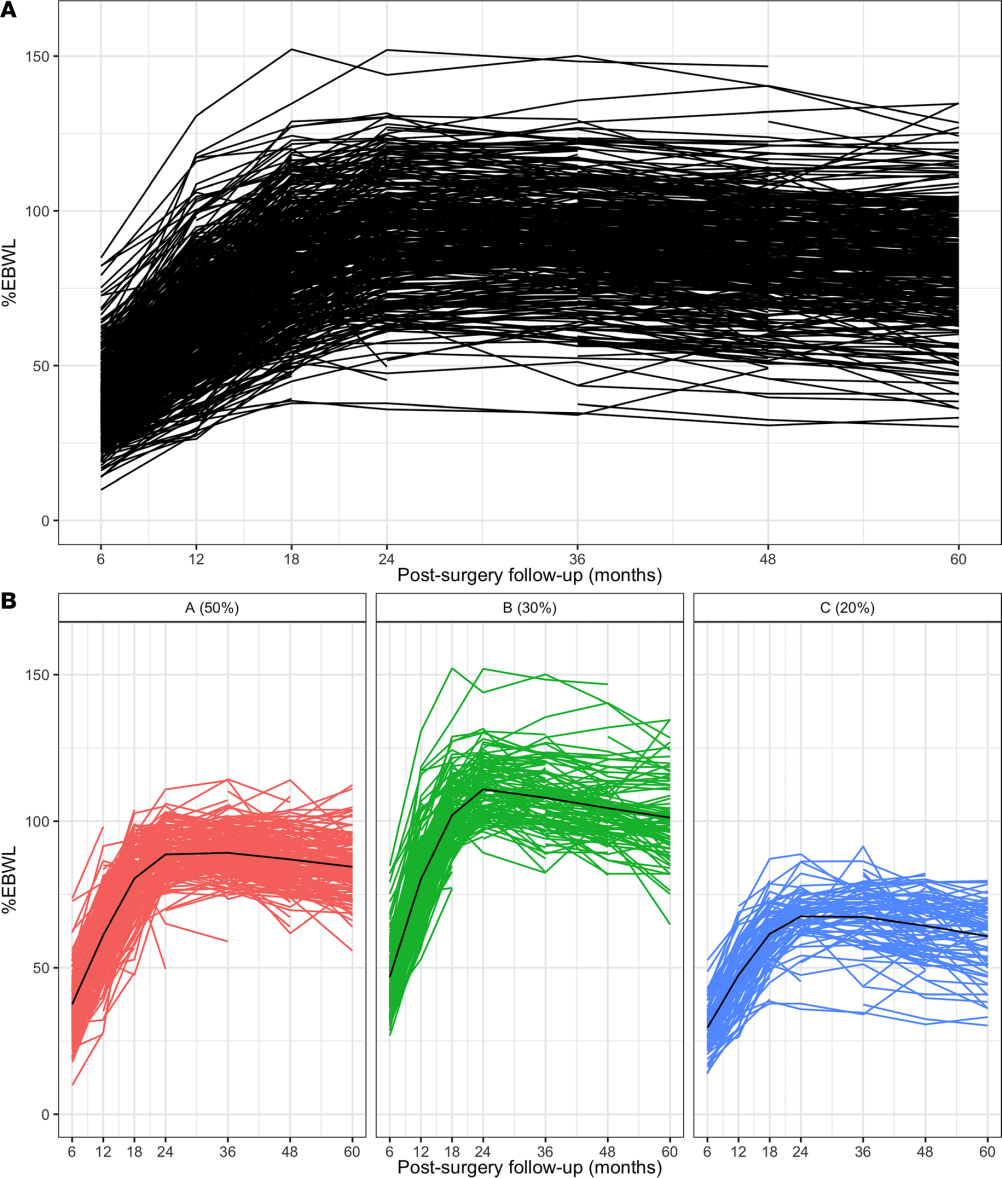
Weight loss trajectories following biliopancreatic diversion with duodenal switch. (**A**) Longitudinal profile of excess body weight loss (%EBWL) during the postsurgery follow-up period for each of the 540 participants of the study. (**B**) Subgroups stand for clusters of participants depending on their weight loss trajectories (low, average, and high) based on %EBWL.

Participants showed varying weight loss responses to bariatric surgery, with a mean %EBWL at the end of the follow-up period of 83.5%, ranging from 30.3% to 134.8% (Table 1 and Figure 1A). Three weight loss trajectory clusters were identified in the study population using the R latrend package, with 262 participants (48.5%) being classified as average responders (74.7 ± 6.0 %EBWL), 168 (31.1%) as high responders (91.2 ± 8.9 %EBWL), and 110 (20.4%) as low responders (56.2 ± 6.9 %EBWL), with mean posterior probabilities of 0.96, 0.92, and 0.93, respectively (Figure 1B).

**Figure 2:**
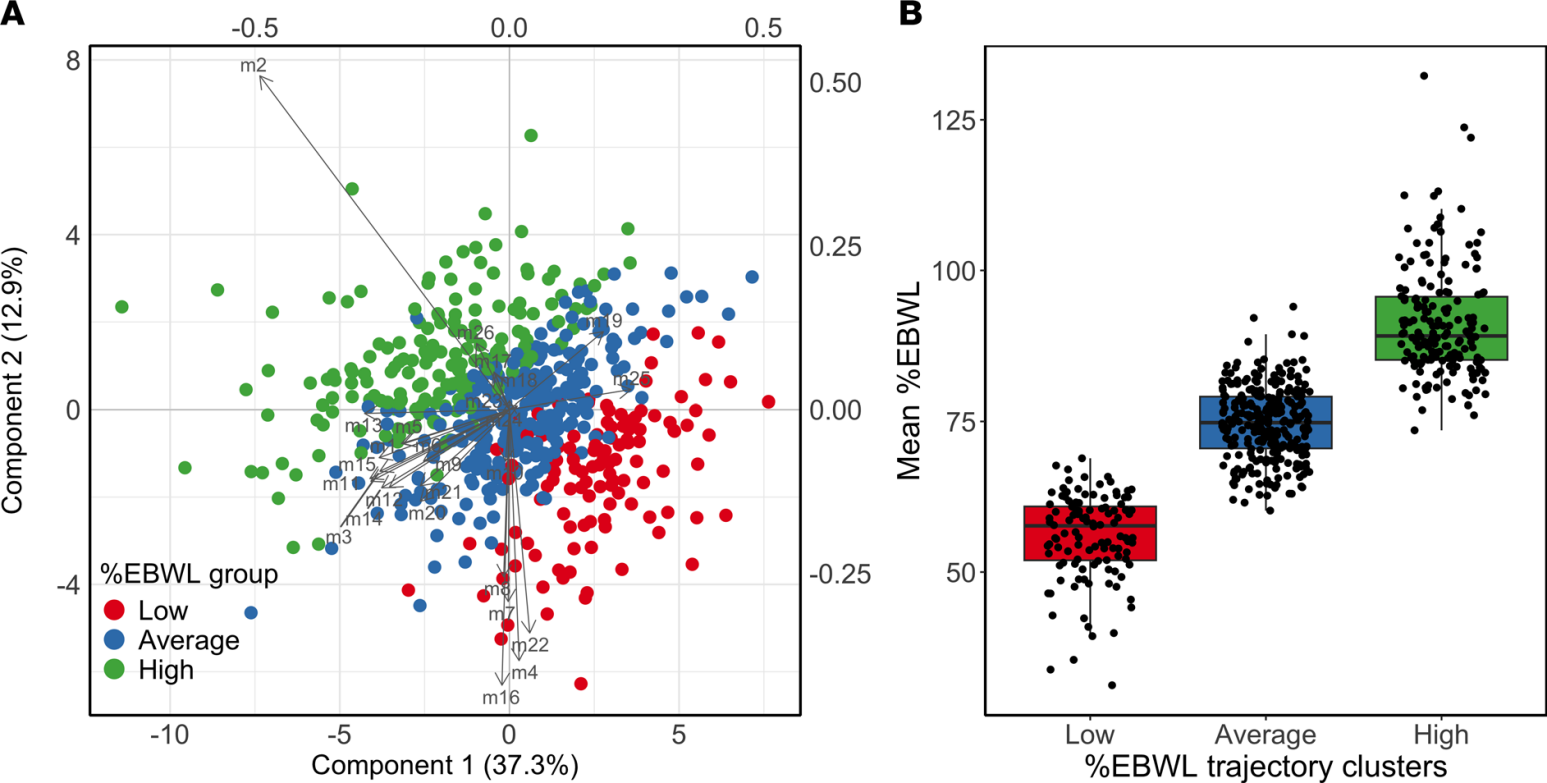
Partial least squares–discriminant analysis (PLS-DA) of excess body weight loss (%EBWL) trajectory parameters. (**A**) Score plot showing the separation of participants based on the 24 longitudinal trajectory parameters derived from the traj analysis, with the percentage of variance explained by the first 2 components. Points are colored according to %EBWL trajectory clusters (low, average, and high). m2 stands for mean-over-time %EBWL (mean %EBWL). (**B**) Distribution of mean %EBWL values across the 3 trajectory clusters. Box-and-whisker plots represent the median and interquartile range, with individual data points overlaid.

A total of 50.2% of the explained variance of %EBWL was attained with the 24 parameters of R traj data (Figure 2A). The mean-over-time %EBWL parameter, hereafter referred to as mean %EBWL, was the measurement better describing the weight loss trajectory clusters (Figure 2A), showing the highest loading weight, and was significantly different among the 3 weight loss trajectory clusters (*P*_ANOVA_ < 0.001) (Figure 2B). Post hoc analysis with Tukey’s honestly significant difference (HSD) test showed significant differences between average and low responders (18.7%, 95% CI = 16.8–20.7, *P*_Tukey_ < 0.001), as well as between the average and high responders (16.6%, 95% CI = 14.9–18.3, *P*_Tukey_ < 0.001). The mean %EBWL difference was greater between low and high responders (35.4%, 95% CI = 33.3–37.5, *P*_Tukey_ < 0.001). The %EBWL at the end of the follow-up period, hereafter referred to as end %EBWL, was also significantly different among the 3 weight loss trajectory clusters (*P*_ANOVA_ < 0.001) (data not shown). These significant differences were observed between the average and the low cluster (23.7%, 95% CI = 20.1–27.4, *P*_Tukey_ < 0.001); between the average and the high cluster (16.7%, 95% CI = 13.2–20.2; *P*_Tukey_ < 0.001), as well as between the low and the high cluster (40.5%, 95% CI = 36.4–44.5, *P*_Tukey_ < 0.001).

### Model performance.

The predictive performance of the null linear model including age, sex, presurgery BMI, type of surgery, and 10 principal components of population structure, but not including PGS, was able to explain 38.5% of mean %EBWL and 18.4% of end %EBWL (data not shown). The inclusion of the PGS constructed in our study population significantly increased the explained variance of mean %EBWL.

**Figure 3:**
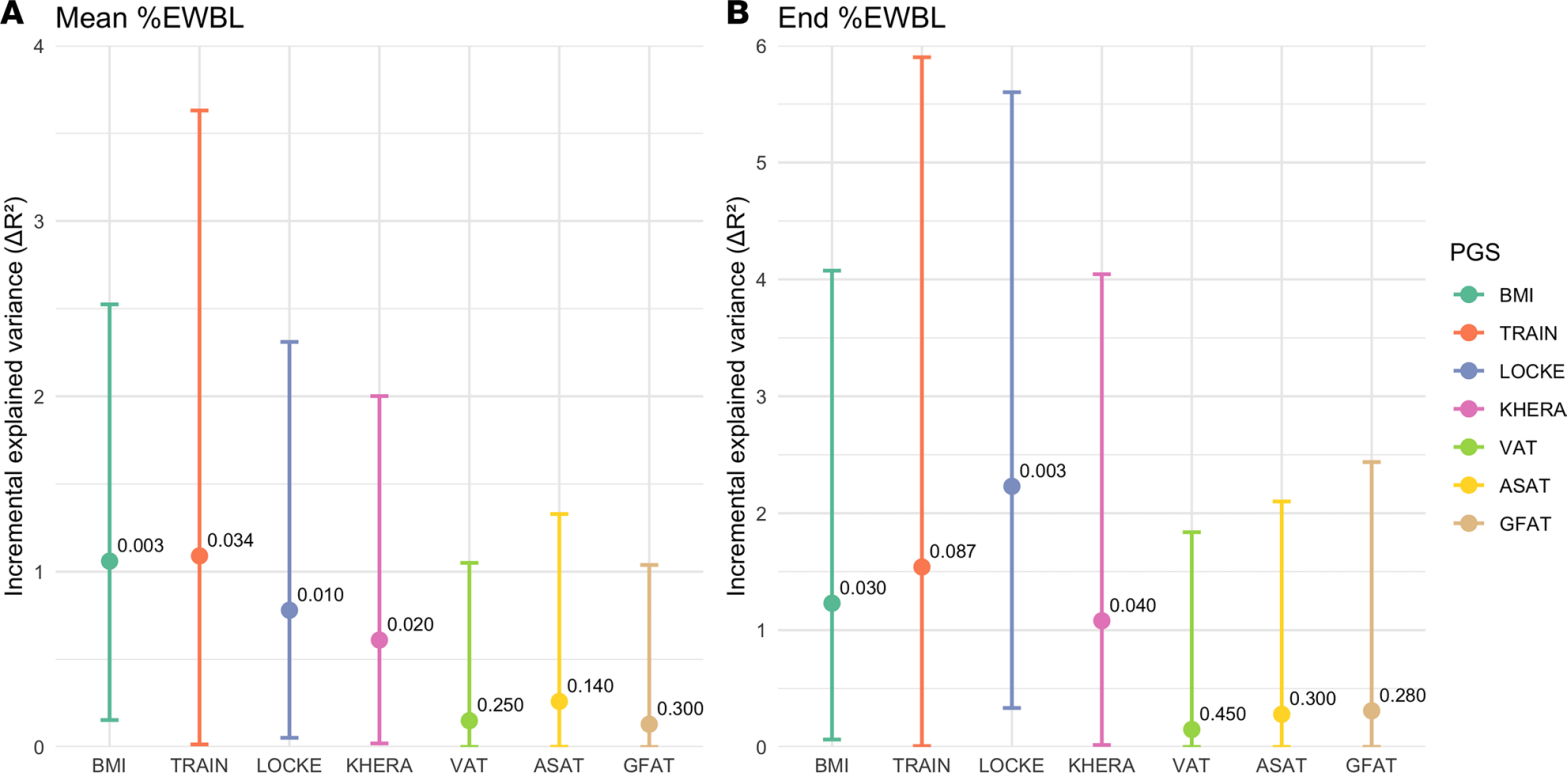
Increase in the explained variance of excess body weight loss (%EBWL) after the inclusion of polygenic risk scores (PGS). (**A**) Shows the incremental explained variance (Δ*R*^2^) for the mean-over-time %EBWL (mean %EBWL), while (**B**) shows the Δ*R*^2^ for %EBWL at the end of follow-up (end %EBWL). Points represent Δ*R*^2^ estimates from multivariable models, adjusted by age, sex, type of surgery, presurgery BMI, and 10 principal components of population structure. Vertical lines indicate 95% CIs derived from 1,000 bootstrap resamples. *P* values correspond to the significance of each PGS when added to the base model.

First, the inclusion of the PGS constructed in the full cohort and based on the BMI GWAS (PGS_BMI_) significantly increased the explained variance of the mean %EBWL by 1.06% (*P* = 0.003), as well as of the end %EBWL by 1.23% (*P* = 0.030) (Figure 3, A and B). Second, the inclusion of the PGS constructed in the training dataset (PGS_TRAIN_) also significantly increased the explained variance of mean %EBWL (1.09%; *P* = 0.034), but not of end %EBWL (1.54%; *P* = 0.087) (Figure 3, A and B). The PGS, constructed for the entire population using a cross-sectional BMI GWAS from an independent cohort of 322,154 individuals (PGS_LOCKE_), explained an additional 0.78% of the variance in mean %EBWL (*P* = 0.01) and 2.23% in end %EBWL (*P* = 0.003) (Figure 3, A and B). Significant results were also obtained when including in the model a PGS constructed in an independent cohort of longitudinal BMI follow-up from birth to 18 years with the same cross-sectional BMI GWAS summary results (PGS_KHERA_). A significant increase of 0.61% (*P* = 00.02) was observed for mean %EBWL and of 1.08% (*P* = 00.04) for end %EBWL (Figure 3, A and B). No significant increase in mean %EBWL or end %EBWL was observed after the inclusion of PGS constructed with summary results from GWAS on visceral (PGS_VAT_), subcutaneous (PGS_ASAT_), or gluteofemoral (PGS_GFAT_) adipose tissue depots (Figure 3, A and B).

**Figure 4:**
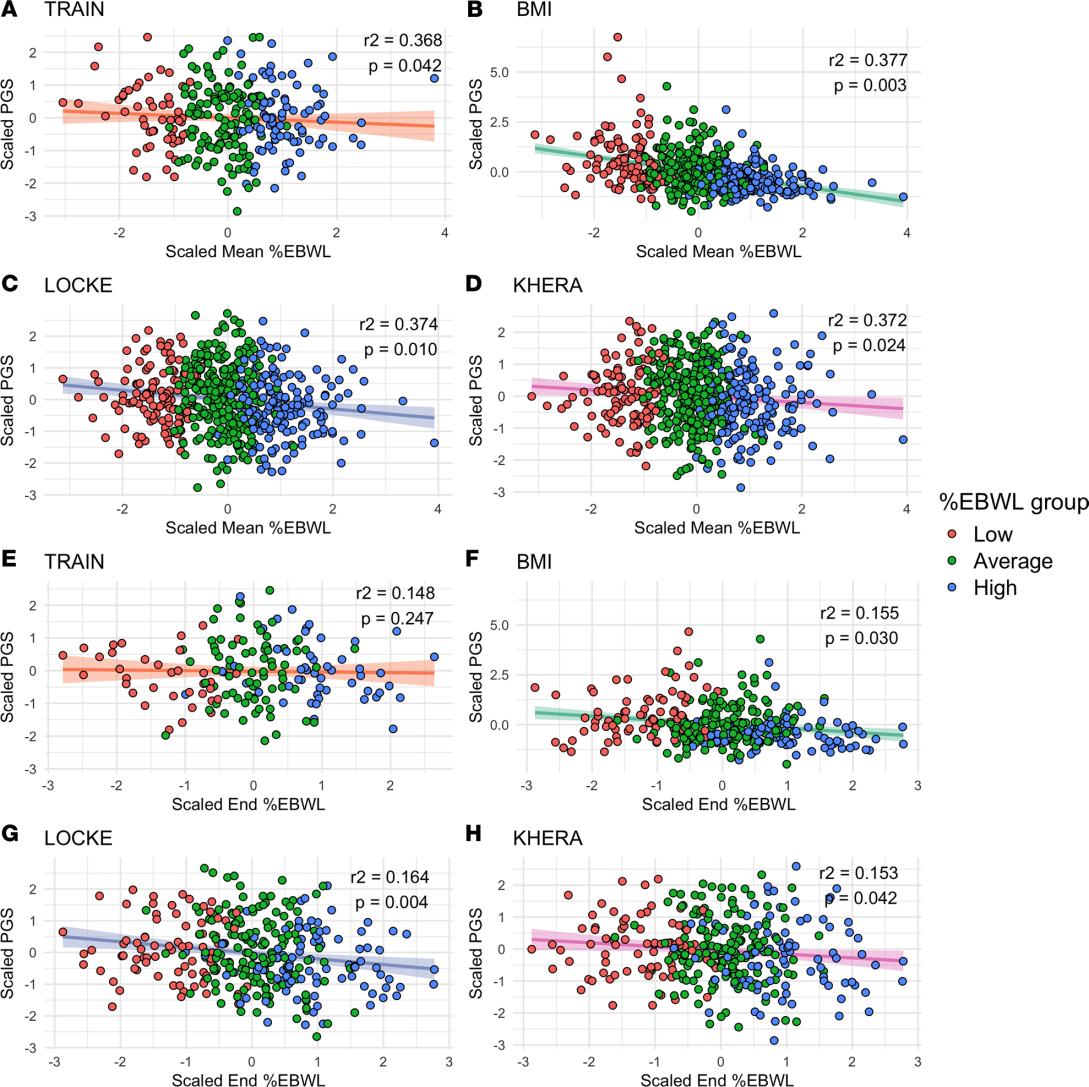
Linear relationship between polygenic risk scores (PGS) and excess body weight loss (%EBWL). (**A**–**D**) Associations with mean-over-time %EBWL (mean %EBWL), and (**E**–**H**) associations with %EBWL at the end of follow-up (end %EBWL). Panels represent individual PGS: (**A** and **E**) PGS_TRAIN_, (**B** and **F**) PGS_BMI_, (**C** and **G**) PGS_LOCKE_, and (**D** and **H**) PGS_KHERA_. Points represent individual participants and are colored according to %EBWL response group (low, average, and high). Solid lines indicate fitted linear regression models adjusted for age, sex, presurgery BMI, surgery type, and the first 10 principal components of population structure, with shaded areas denoting 95% CIs. Reported *r*^2^ and *P* values correspond to the association between each PGS and %EBWL.

The linear relationship of the PGS showing a significant impact on %EBWL variance explanation (PGS_BMI_, PGS_TRAIN_, PGS_LOCKE_, and PGS_KHERA_) were further tested for mean and end %EBWL. As shown in Figure 4, all the analyzed PGS showed a significant and negative association with both mean and end %EBWL, except for PGS_TRAIN_ with end %EBWL (*P* = 00.085). The relationship was consistently stronger for mean %EBWL (*r*^2^ ≈ 0.4) than for last recorded %EBWL (*r*^2^ ≈ 0.2), together with a higher degree of data completeness for mean %EBWL (540/540; 100%), as compared with last recorded %EBWL (328/540; 60%).

### Classification accuracy.

**Figure 5:**
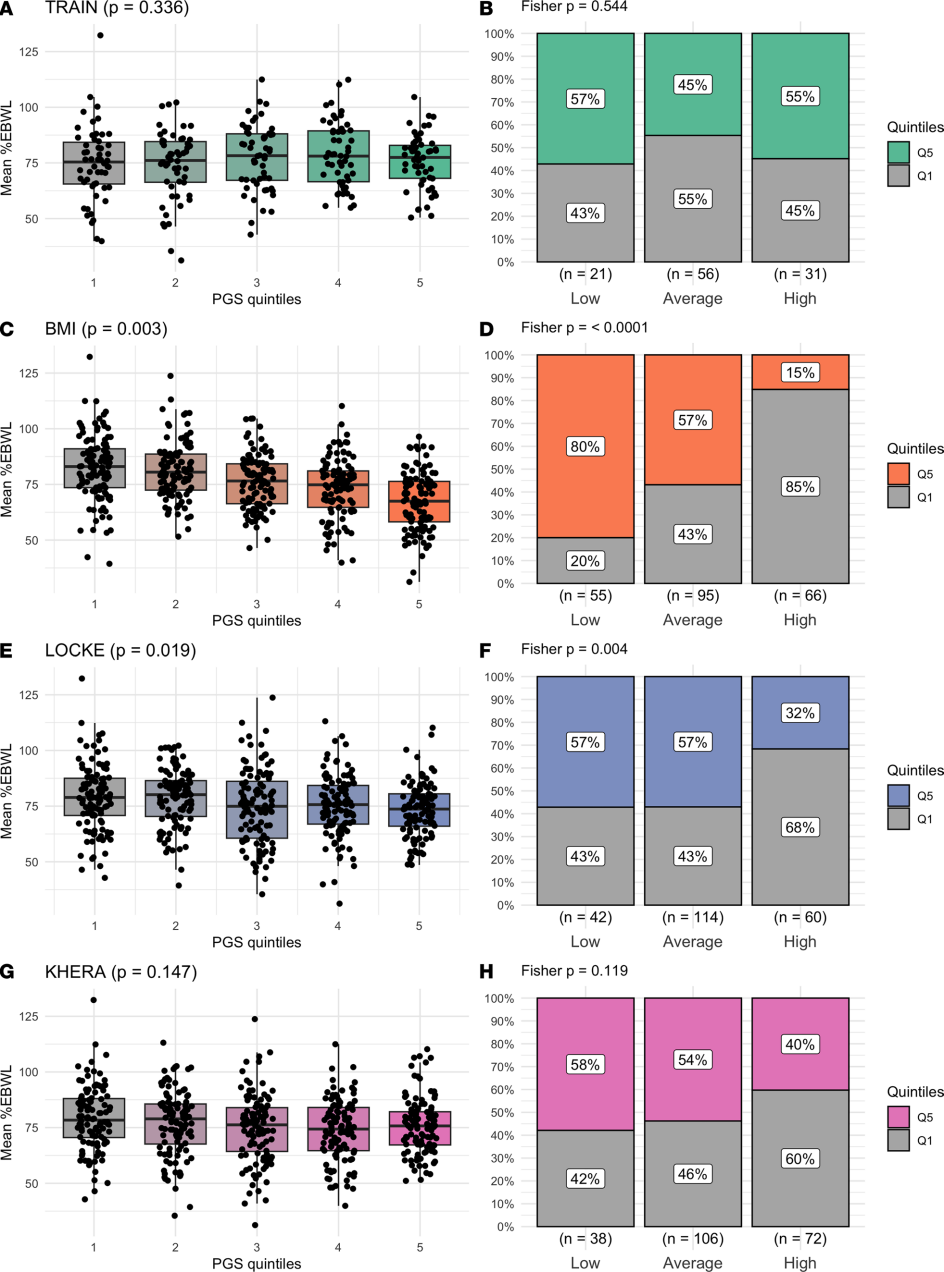
Predictive capacity of polygenic risk scores (PGS) to classify participants according to their excess body weight loss (%EBWL). Left panels show the distribution of mean-over-time %EBWL (mean %EBWL) across quintiles of each PGS, while right panels display the proportion of participants classified into %EBWL response subgroups (low, average, and high) within each PGS quintile category. (**A** and **B**) PGS_TRAIN_. (**C** and **D**) PGS_BMI_. (**E** and **F**) PGS_LOCKE_. (**G** and **H**) PGS_KHERA_. Box-and-whisker plots represent the median and interquartile range, with individual data points overlaid. Stacked bar plots illustrate the percentage of participants within each %EBWL response subgroup. Fisher’s exact test *P* values assessed differences in response distribution across PGS quintiles.

The ability of a PGS to correctly classify participants according to its expected mean %EBWL subgroup was finally tested with the PGS expressed into quintiles. Among the PGS constructed in our study population, PGS_TRAIN_ did not show a significant association (*P* = 0.336) (Figure 5A, see also Figure 5B), whereas PGS_BMI_ showed a significant association with mean %EWBL (*P* = 0.003) (Figure 5C). Participants within the fifth PGS_BMI_ quintile, i.e., with a high risk of low %EBWL response, showed a mean %EBWL decrease as compared with those in the first quintile of around 15% (82.4 ± 15.1 vs. 67.5 ± 13.2 %EBWL) (Figure 5C). The proportion of participants within each response subgroup was significantly different according to the first and fifth PGS_BMI_ quintiles (*P*_Fisher_ = 3.1 × 10^–13^), with 80% of participants in the fifth quintile being classified as low responders and 57% as average responders, while only 15% was classified as high responders (Figure 5D). The risk ratio (RR) between low and average subgroups was significantly different (RR = 3.4, 95% CI = 1.5–7.7), as well as between low and high subgroups (RR = 9.7, 95% CI = 4.5–20.7). Similar results were obtained with PGS_LOCKE_, also developed in our study population but with GWAS data from an independent cohort. A significant association was found with mean %EBWL through the PGS_LOCKE_ quintiles (*P* = 0.019) (Figure 5E, see also Figure 5F), with a mean decrease between the first and the fifth quintile of around 5% (78.9 ± 14.6 vs. 73.6 ± 12.2 %EBWL). The proportion of participants within the highest PGS_LOCKE_ quintile in low- and average-response subgroups was 57%, while it significantly decreased to 32% in the high-response subgroup (*P*_Fisher_ = 0.025), with a significant RR between low- and high-response subgroups (RR = 1.6, 95% CI = 1.1–2.4). Finally, no association between PGS_KHERA_ and mean %EWBL was found (*P* = 0.147) (Figure 5G, see also Figure 5H).

## Discussion

A longitudinal follow-up of patients who underwent bariatric surgery was previously conducted to monitor the progression of their body weight change over a 5-year period (11). In the present study, we identified the most representative parameters of the weight loss trajectories through partial least squares–discriminant analysis (PLS-DA). Out of the 24 parameters identified, mean %EWBL stood out as the best driving parameter, and was therefore retained as the primary outcome to compute PGS of the weight loss response. Last recorded %EWBL, or end %EBWL, was also retained as secondary endpoint. PGS were then benchmarked using GWAS data from other independent studies.

As expected, the PGS constructed herein are much stronger predictors of weight loss than of other adiposity traits, namely VAT, SAT, and GFAT. This observation aligns with the fact that our GWAS, along with models from Locke et al. (10) and Khera et al. (12), but not from Argawal et al. (13), was constructed based on BMI. Despite that mean %EBWL accounted for the largest proportion of variance among all 24 parameters, adding the PGS to the model relatively increased the explained variance of end %EBWL in a more important way. Since mean %EBWL has greater effect size than end %EBWL, its impact on the PGS was expectedly more substantial. This is consistent with the more significant associations found between all PGS tested with mean %EBWL, than with last recorded %EBWL, with similar coefficients of determination across all PGS models. A direct comparison of our study with that from de Toro-Martín et al. (8) is challenging due to differences in patient cohorts, as well as the metrics and algorithms employed to classify participants, making it difficult to draw definitive conclusions about the additional benefits of the genome-wide PGS presented here compared to those derived from a limited number of SNPs. In the previous study (8), we utilized a PGS consisting of 186 SNPs that were significantly associated with BMI based on a prior GWAS by Locke et al. (10). This approach led to a significant yet moderate enhancement in predictive accuracy for the weight loss response to the bariatric surgery, demonstrated by an increase in AUC of 0.021 compared with the full model without the PGS, which corresponds to a relative improvement of approximately 2.1%. Although this relative improvement appears be within a range that is comparable to the increases in %EBWL explained variance observed in the current study, ranging from 0.61% to 2.23%, it is essential to recognize that AUC and explained variance are fundamentally distinct metrics. While AUC assesses a model’s capacity to discriminate between positive and negative cases, explained variance measures the proportion of outcome variability explained by the model. Consequently, direct comparisons between these metrics should be approached with caution. Nonetheless, the modest increase in %EBWL explained variance observed here is consistent with findings from recent studies evaluating cross-trait PGS for complex traits, where reported values are often around 1% (14). This limited improvement aligns with the polygenic nature of traits like %EBWL, where numerous genetic loci contribute with small individual effects (10).

Moreover, the PGS accurately classified participants into the 3 subgroups of weight loss trajectories across all studies. The choice of this multinomial metric also aimed at reflecting a higher precision compared with the binomial data previously used and derived from regrouping participants into 2 responder types (8), which may be viewed as a limitation. To illustrate these results, the significant RR between low and high responders in PGS indicates that individuals in the fifth quintile (those with presumably more favorable genetic predisposition for weight loss) are more likely to have a high response to weight loss surgery (higher mean %EBWL) compared with those in the first quintile (those with less favorable genetic predisposition). These results also point to a strong effect of the genetic factors captured by the PGS on surgery outcomes. While it remains uncertain whether SNPs included in the PGS are linked to traits of weight loss, such as metabolic rate, fat storage, and appetite regulation, the high RR indicates that they are effective predictors of the likelihood of benefiting from surgery. This aligns with the precision medicine paradigm and supports the potential use of PGS as clinical tool to predict patient outcomes for weight loss surgery and guide treatment decisions. For instance, individuals with a high PGS might be prioritized for surgery or provided with different postoperative management strategies compared with those with a low score. Low-score patients could be redirected toward more conservative treatments like medication to avoid unnecessary drawbacks.

An important element to be taken into account to ensure that future PGS are equally applicable at population levels is that they must be built from diverse genetic backgrounds. In the present study, the population was entirely of European ancestry, which currently limits the generalizability of the findings. In this sense, replication of PGS in independent cohorts from diverse populations should be considered in future research. Herein, PGS performance was evaluated using an internal train-test split rather than an entirely independent external cohort. Although this approach reduces overfitting relative to using a single dataset, it may still lead to optimistic estimates of predictive performance due to shared characteristics between training and test samples. Again, replication of PGS in independent cohorts, ideally drawn from diverse populations, clinical settings and other types of bariatric surgery (e.g., Roux-en-Y or gastric sleeve) should be considered in future research before considering their potential clinical utility. Moreover, although PGS were significantly associated with weight loss outcomes after bariatric surgery, it is worth emphasizing that the incremental proportion of explained variance attributable to PGS remained modest. While PGS capture a measurable genetic contribution, they are unlikely to be sufficient as standalone predictors at the individual level. Instead, their primary value may lie in contributing to multivariable risk stratification frameworks that integrate clinical, surgical, behavioral, and genetic factors, rather than serving as independent clinical decision tools.

Several studies have explored the potential of using genetic tools to predict weight loss response to bariatric surgery. Ciudin et al. (15) developed a PGS based on 50 genotyped SNPs from 39 genes previously associated with weight loss following bariatric surgery and lifestyle intervention. %EBWL at nadir was measured in 416 patients who underwent bariatric surgery. The model, including age, type of surgery, presence of diabetes, and PGS reached an area under the ROC curve of 0.845 (95% CI = 0.805–0.880) (15). In another study by Thanos et al. (16) the authors verified whether %EBWL could be predicted in 30 patients via genetic addiction risk severity score, a genetic test designed to assess an individual’s genetic predisposition to addiction and substance use disorders. They found that the risk severity score was positively correlated with %EBWL (Spearman’s correlation [*r*_s_] = 0.424, 95% CI = 0.056–0.690), weight change (*r*_s_ = 0.397, 95% CI = 0.024-0.673), and BMI change (*r*_s_ = 0.378, 95% CI = 0.002–0.660) (16). Interestingly, an increase in incidence of post–bariatric surgery onset of alcohol and substance use disorder has been documented (17). One hypothesis to explain this observation is that the surgery induces changes in the expression of genes involved in the reward circuitry, further emphasizing the importance of genomic research in bariatrics (17).

Recently, Mas-Bermejo et al. used a candidate gene approach to build a PGS using 7 SNPs in 5 obesity-related genes, namely *FTO*, *MC4R*, *SIRT1*, *LEP*, and *LEPR*, to predict weight loss after bariatric surgery in 104 patients (18). They observed significant associations between the PGS and %EBWL (*P* = 1.5 × 10^–5^), total weight loss (*P* = 3.1 × 10^–8^), and BMI change (*P* = 7.8 × 10^–16^) after 60 months (18). Similarly, Peña et al. used 50 common SNPs previously associated with different obesity phenotypes and from GWAS data meta-analysis to build a PGS of the weight loss response to bariatric surgery in 106 patients (19). The sole PGS was significantly associated with total weight loss (*P* = 0.009) and BMI change (*P* = 0.009) (19).

Although these studies strongly support the hypothesis that genetic variants play an important role in the variability observed in weight loss following bariatric surgery and that PGS could be a valuable clinical tool, there is a lack of consistency in the selection of genes and SNPs, which are often chosen using a candidate-gene approach. There are contradictions in the results regarding which SNPs and genes are associated with the weight loss response as well. In a recent review by Pereira et al. (20), the authors suggest that although multiple genes like *FTO*, *POMC*, *MC4R*, *LEP*, and *LEPR* have repeatedly been associated with the weight loss response, the discrepancy in the literature currently makes the creation of effective prediction tools impossible in the short term. Since the weight loss response is likely to be very polygenic in nature, the candidate gene approach may be limited by its tendency to overlook certain genomic regions. An important advantage of using a genome-wide method, as done in the present study, is that it allows bypassing disparities in the literature on optimal SNP selection for PGS building. By using an agnostic approach to identify SNPs, we were able to select them based on statistical relevance across the whole genome and we were thus able to build a PGS that reached a relatively high predictive capacity of %EBWL. This supports the notion that PGS implementation in multivariable risk stratification frameworks of clinical practice could actually be possible in a not-too-distant future. Findings from Khera et al. corroborate this assumption (12). They built a PGS of the susceptibility to obesity with relatively high predictive capacity for its prevalence (12). After stratifying the population into deciles of PGS score, obesity was present in 43.2% of individuals in the top decile, compared with 9.5% in the bottom decile (12). The risk of severe obesity was 25 times higher in individuals in the top decile (*P* < 0.0001) (12). These promising results clearly demonstrate the advantage of using a genome-wide method for building clinical tools for predicting features that are influenced by genetics such as obesity.

In conclusion, findings of the present study provide strong evidence supporting the potential of PGS as predictive tools for bariatric surgery outcomes, emphasizing the importance of precision treatment strategies. The promising predictive performance obtained underscores the significant influence of genetic factors on the response to medical interventions, paving the way for more targeted and effective antiobesity treatments based on genetic profiling. However, further validation is needed to develop precise and cost-effective tools for clinical applications, and future studies integrating PGS with health economic models will be necessary to evaluate their potential impact on clinical decision-making. Additional research in more diverse populations is essential to ensure the generalizability and equity of these predictive tools.

## Methods

### Sex as a biological variable.

This study involved human participants of both sexes. Sex was included as a biological variable in the statistical analyses to account for confounding variables. No sex- or gender-specific analysis or stratification was conducted beyond this adjustment, including during participant selection.

### Participant selection.

An initial cohort of 565 patients who underwent biliopancreatic diversion with duodenal switch was considered for the present study (11) (Supplemental Table 1). Population structure was assessed by principal component analysis (PCA) using a reference-based approach implemented in bigsnpr, following the previously described bedpca framework (21). Study participants were projected onto principal components derived using external reference populations from the UK Biobank, comprising 21 genetically defined ancestry groups (22). These reference groups were subsequently consolidated into broader ancestry categories corresponding to those observed in the study population (European, South American, East African, and Middle Eastern). The study population was predominantly of European genetic ancestry (558 individuals, 98.7%), with a small number of participants of South American (*n* = 5, 0.9%), Middle Eastern (*n* = 1, 0.2%), and East African (*n* = 1, 0.2%) ancestry (Supplemental Figure 1). To maintain a genetically homogeneous study cohort, participants with non-European ancestry were excluded from further analyses (*n* = 7). Genetic relatedness was evaluated using the KING-robust kinship estimator implemented in PLINK and applied through the bigsnpr R package. Based on kinship coefficients, 5 pairs of first-degree relatives (kinship coefficient ~0.25) and 1 pair of second-degree relatives (~0.125) were identified, leading to the exclusion of 12 individuals to ensure sample independence. PCA-based outlier detection was subsequently performed using distance and outlierness statistics as described in the bedpca methodology (23) to identify individuals with atypical genetic profiles potentially reflecting genotyping artifacts or population heterogeneity. Six participants exceeding the predefined outlierness threshold were excluded from further analyses (Supplemental Figure 2). After applying relatedness filtering, PCA-based outlier exclusion, and ancestry-based exclusions, a final sample of 540 participants was retained for all statistical analyses (Table 1).

### Intervention.

The complete intervention was previously described (11). Briefly, all 540 patients presented severe obesity (BMI ≥ 35 kg/m^2^) and underwent biliopancreatic diversion with duodenal switch between February, 2008 and March, 2015 at the Bariatric Surgery Clinic of the Quebec Heart and Lung Institute. One of the 2 following types of surgery was used for each patient: open surgery or laparoscopy. Fasting blood samples for measurement of metabolic parameters and DNA isolation were taken preoperatively. Height and body weight were measured 1 day prior to surgery. Further body weight measurements were taken during postoperative visits or phone calls for 5 years at time points of 3, 6, 12, 24, 36, 48, and 60 months to track body weight change. %EBWL was then calculated as the ratio between actual weight loss (initial BMI minus time-point BMI) to ideal body weight loss (initial BMI minus ideal BMI, fixed at 25 kg/m^2^), multiplied by 100. All participants had data for at least 4 of the 7 time points following the surgery. Blood samples for DNA extraction were obtained from the Biobank of the Quebec Heart and Lung Institute according to institutionally approved management modalities.

### Clustering.

Participants were classified according to their weight loss achievement over 5 years following bariatric surgery. Participant classification into weight loss trajectories was done using a nonparametric trajectory longitudinal *k*-means (KML) clustering method implemented in the R latrend package (24) and using %EBWL at 6, 12, 18, 24, 36, 48, and 60 months after the bariatric surgery as input parameters. The final number of clusters was chosen based on BIC minimization while keeping mean posterior probabilities over 0.90. The R traj package v1.3.1 (25) was used to calculate 24 measures describing the features of the longitudinal trajectories. A PLS-DA implemented in mixOmics v6.22.0 (26) was used to determine those parameters maximizing the differences between weight loss trajectory clusters to be selected for testing PGS performance.

### Genotyping.

The Global Diversity Array-8 Kit v1.0 (GDA) BeadChip was used to genotype the study population. The GDA array provides targeted coverage of more than 4,800 key genes across the genome. Approximately 1.8 million markers are included on the BeadChip for high exonic coverage in regions of disease relevance, providing highly accurate copy number variation calls, and an average resolution of 1.5 Mb. The study population was randomly partitioned into training and test datasets in a 1:1 ratio using a stratified sampling approach to preserve the distribution of weight loss trajectory clusters. Genotype quality control was performed separately for the full cohort (*n* = 540) and the training subset (*n* = 270) using PLINK v1.9 (https://www.cog-genomics.org/plink/); a total of 1,717,562 SNPs were initially available for GWAS. SNPs were filtered based on missing genotype rate (>10%; --geno 0.1), deviation from Hardy-Weinberg equilibrium (HWE) (*P* < 1 × 10^–50^; --hwe 1 × 10^–50^), and minor allele frequency (MAF < 1%; --maf 0.01). Individuals with more than 10% missing genotypes were excluded (--mind 0.1), although no individuals were removed based on this criterion. For the full cohort, 1,717,562 SNPs were initially available, with an overall genotyping rate of over 99.8%. After quality control, 2,822 SNPs were removed due to missingness, 2 due to HWE, and 907,220 due to low MAF, resulting in 807,518 SNPs retained for GWAS. For the training dataset, 2,940 SNPs were removed due to missingness and 912,467 due to low MAF, with no SNPs excluded for HWE. This resulted in 802,155 SNPs retained for GWAS.

### GWAS.

Two unadjusted linear GWAS of presurgery BMI were performed using PLINK to generate summary statistics for PGS construction, in both the full cohort and the training dataset. For each GWAS, summary statistics included effect size estimates (β), standard errors, and *P* values, which were subsequently used for PGS derivation. No variants reached the genome-wide significance threshold in any of the GWAS (Supplemental Figure 3). Genomic inflation was minimal across all GWAS, with genomic control inflation factors (λGC) values of 0.999 in the full cohort and 0.993 in the training dataset, indicating no evidence of systematic inflation of association statistics (Supplemental Figure 3).

### PGS construction.

The analysis of the massive SNP array was performed with the R package bigsnpr v1.12.6 (27). LDpred-2 was also used to infer genetic architecture parameters with LDpred2-auto (27). PRSice-2 was used for calculating, applying, and evaluating the PGS results (28). We used sex, age, presurgery BMI, surgery type, and 10 principal components of population structure as covariates in the PGS models. The construction of BMI-based PGS was performed using 2 continuous outcomes derived from the trajectory analysis: mean %EBWL, defined as the average percentage of excess body weight loss across all available follow-up time points, and end %EBWL, defined as the percentage of excess body weight loss at the final observed follow-up time point for each participant.

### PGS benchmarking.

Summary statistics from previous GWAS were used for benchmarking the performance of the different PGS constructed in the present study. After human genome version conversion with the UCSC LiftOver tool (https://genome.ucsc.edu/cgi-bin/hgLiftOver), summary statistics from previous studies were standardized to the following headers: SNP identifier, chromosome and base pair position, common and rare allele, and β value from GWAS and MAF. An overview of studies is presented in Table 2. First, we built PGS models using statistics from Locke et al. (10), a study combining GWAS and Metabochip meta-analysis of BMI in 339,224 individuals. After SNP matching and genome conversion, 2,547,979 SNPs were retained. Second, statistics from 3 GWAS performed by Agrawal et al. (13) on VAT, SAT, and GFAT fat depots were also used to construct respective PGS models. A total of 11,485,690 SNPs in 38,965 individuals were retained. Third, the performance of a previous PGS of BMI constructed by Khera et al. (12) was also tested. This PGS is based on GWAS summary statistics form Locke et al. (10) and included 2,100,301 SNPs in more than 119 951 individuals ranging from middle age to birth.

### Statistics.

All statistical analyses were performed in R. The latrend package was used for clustering of longitudinal %EBWL trajectories with *k*-means at 7 time points over 5 years, and features were quantified using the traj package. PLS-DA was performed to identify the trajectory parameters that best discriminated participants according to their %EBWL trajectory clusters (low, average, and high). The analysis was conducted using the 24 parameters derived from the traj modeling, with clustering used as the outcome. Differences in %EBWL trajectory clusters were assessed using 1-way ANOVA, with post hoc comparisons conducted using Tukey’s HSD test. The distribution of mean %EBWL categories across quintiles of PGS was evaluated using Fisher’s exact test, and RRs were calculated from observed proportions. Linear models were adjusted for sex, age, presurgery BMI, surgery type, and 10 principal components of population structure, and 95% CIs were obtained using 1,000 bootstrap resamples. The partition of the study population was performed using the createDataPartition function from the caret R package, with a 50:50 proportion while preserving the distribution of weight loss trajectory clusters across subsets. Statistical significance was set at a *P* value of less than 0.05.

### Study approval.

This study was conducted accordingly to the Declaration of Helsinki and received approval from the ethics committees of the Université Laval and the Quebec Heart and Lung Institute. All participants provided oral and written informed consent before they were enrolled in the study.

### Data availability.

Values for all data points in graphs are reported in the Supporting Data Values file.

## Author contributions

BVM co-wrote the manuscript and interpreted the data. JDTM performed statistical analysis, interpreted the data, and co-wrote the manuscript. BVM and JDTM are both co–first authors of the manuscript. The authorship order was decided upon mutual agreement based on contribution to the writing process. MCV conceived and designed the research. AT and LP participated in the elaboration of the study design. SM participated in clinical care of patients, patient recruitment, and blood sampling. All authors thoroughly read and reviewed the manuscript.

## Conflict of interest

AT receives funding from Johnson & Johnson, Medtronic, and Biotwin for studies on obesity and bariatric surgery. AT acted as consultant for Novo Nordisk, Eli Lily, and Biotwin.

## Funding support

- Canadian Institutes of Health Research (grant PJT-168876).
- Fonds de Recherche du Québec-Santé doctoral studentship (to BVM).
- Fonds de Recherche du Québec-Santé postdoctoral fellowship (to JDTM).

## Supplementary Material

Supplemental data

Supporting data values

## Figures and Tables

**Table 1:**
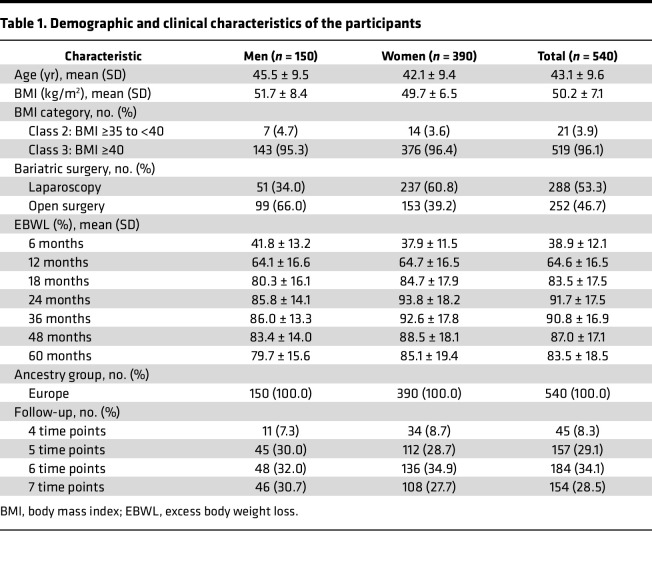
Demographic and clinical characteristics of the participants

**Table 2:**
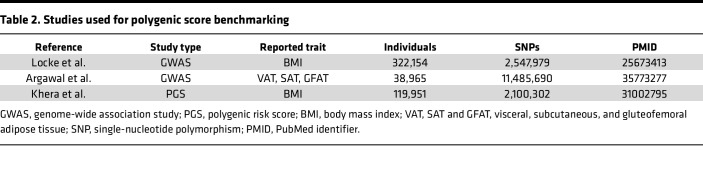
Studies used for polygenic score benchmarking

## Version 1 03/23/2026

In-Press Preview

## Version 2 05/08/2026

Electronic publication
