# Supplementary material for: Agnostic polygenic prediction of weight loss after bariatric surgery
Source: JCI Insight. 2026 Mar 19;11(9):e198133. doi: 10.1172/jci.insight.198133 (PMC13232012; doi:10.1172/jci.insight.198133)
Supplement: Supplemental data [file jciinsight-11-198133-s012.pdf]

Agnostic polygenic prediction of weight loss after bariatric surgery

Bastien Vallée Marcotte[1,2], Juan de Toro-Martín[1,2], André Tchernof[1,2,3], Louis Pérusse[1,4], Simon Marceau[3,5], Marie-Claude Vohl[1, 2]*

1. Centre Nutrition, santé et société (NUTRISS) - Institut sur la nutrition et les aliments fonctionnels (INAF), Université Laval, Quebec City, Quebec, Canada.
2. School of Nutrition, Université Laval, Quebec City, Quebec, Canada.
3. Quebec Heart and Lung Institute Research Centre, Laval University, Quebec, Quebec, Canada.
4. Department of Kinesiology, Faculty of Medicine, Université Laval, Québec, Québec, Canada.
5. Department of Surgery, Faculty of Medicine, Université Laval, Québec, Canada.

*Corresponding author:

Marie-Claude Vohl, Ph.D.
Centre NUTRISS
2440 Hochelaga Blvd.
Quebec, QC, Canada
G1V 0A6
Tel.: (418) 656-2131 ext. 404676, Fax: (418) 656-5877
E-Mail: marie-claude.vohl@fsaa.ulaval.ca

Bastien Vallée Marcotte and Juan de Toro-Martín are co–first authors.

Table S1. Demographic and clinical characteristics of the participants prior to participant exclusion.

Characteristic	Men (n=156)	Women (n=409)	Total (n=565)
Age (yr) — mean (SD)	45.5±9.4	42.2±9.5	43.1±9.6
BMI (kg/m^2) — mean (SD)	51.7±8.3	49.7±6.5	50.2±7.1
BMI category — no. (%)			
Class 1: BMI ≥30 to <35	0 (0.0)	1 (0.2)	1 (0.2)
Class 2: BMI ≥35 to <40	7 (4.5)	13 (3.2)	20 (3.5)
Class 3: BMI ≥40	149 (95.5)	395 (96.6)	544 (96.3)
Bariatric surgery — no. (%)			
Laparoscopy	51 (32.7)	244 (59.7)	295 (52.2)
Open surgery	105 (67.3)	165 (40.3)	270 (47.8)
EBWL (%) — mean (SD)			
6 months	41.6±13.0	37.7±11.4	38.8±12.0
12 months	63.9±16.5	64.7±16.6	64.5±16.5
18 months	80.0±16.1	84.5±17.9	83.3±17.6
24 months	85.2±14.8	93.8±18.1	91.6±17.7
36 months	85.6±13.9	92.4±17.8	90.5±17.1
48 months	82.9±14.6	88.5±18.2	86.9±17.4
60 months	79.4±16.1	85.0±19.3	83.4±18.6
Ancestry group — no. (%)			
Europe	154 (98.8)	404 (98.8)	558 (98.7)
South America	1 (0.6)	4 (1.0)	5 (0.9)
East Africa	0 (0.0)	1 (0.2)	1 (0.2)
Middle East	1 (0.6)	0 (0.0)	1 (0.2)
Follow-up — no. (%)			
4 time-points	17 (10.9)	45 (11.0)	62 (11.0)
5 time-points	50 (32.1)	127 (31.1)	177 (31.3)
6 time-points	47 (30.1)	141 (34.5)	188 (33.3)
7 time-points	42 (26.9)	96 (23.5)	138 (24.4)

BMI, body mass index; EBWL, excess body weight loss.

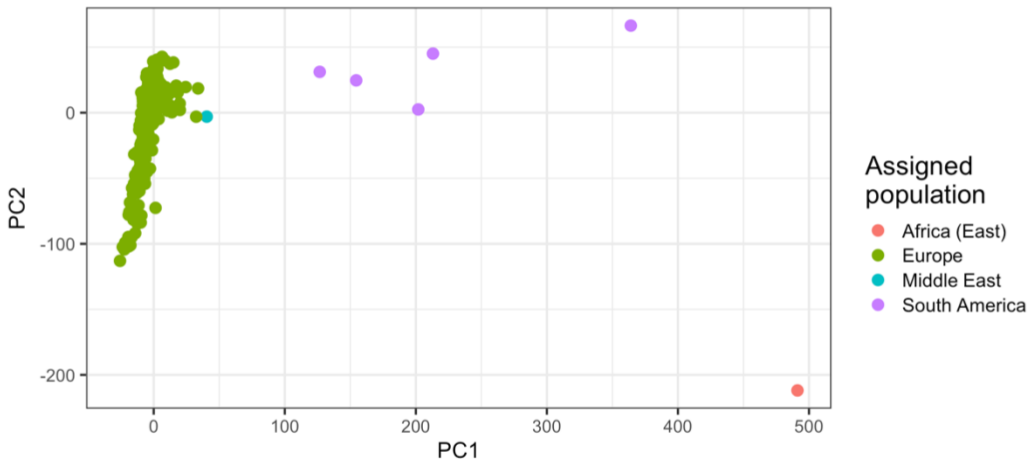

Figure S1. PCA projection and ancestry assignment using UK Biobank reference populations. Study participants were projected onto a principal component (PC) space defined using external reference populations, and each individual was assigned to the closest reference population based on squared distance to reference population centers in PC space. Points show PC1 versus PC2 and are colored by the assigned population group. Individuals assigned to South America, Africa (East), or Middle East were excluded from downstream analyses.

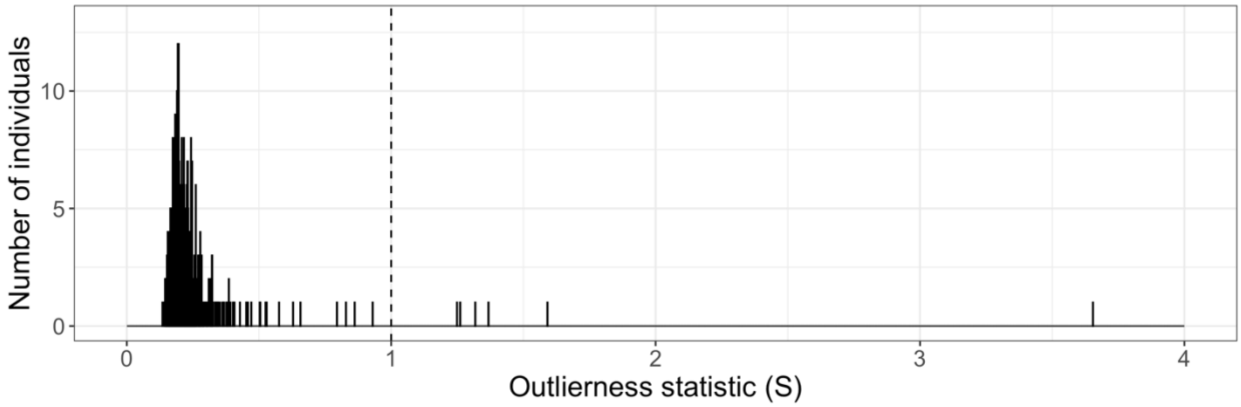

Figure S2. Identification of genetic outliers by principal component analysis. Histogram derived from quality-controlled genotype data. The outlierness statistic (S) quantifies deviation from the main population cluster in PCA space. Individuals with S>1 (n=6) were classified as genetic outliers and excluded from downstream analyses

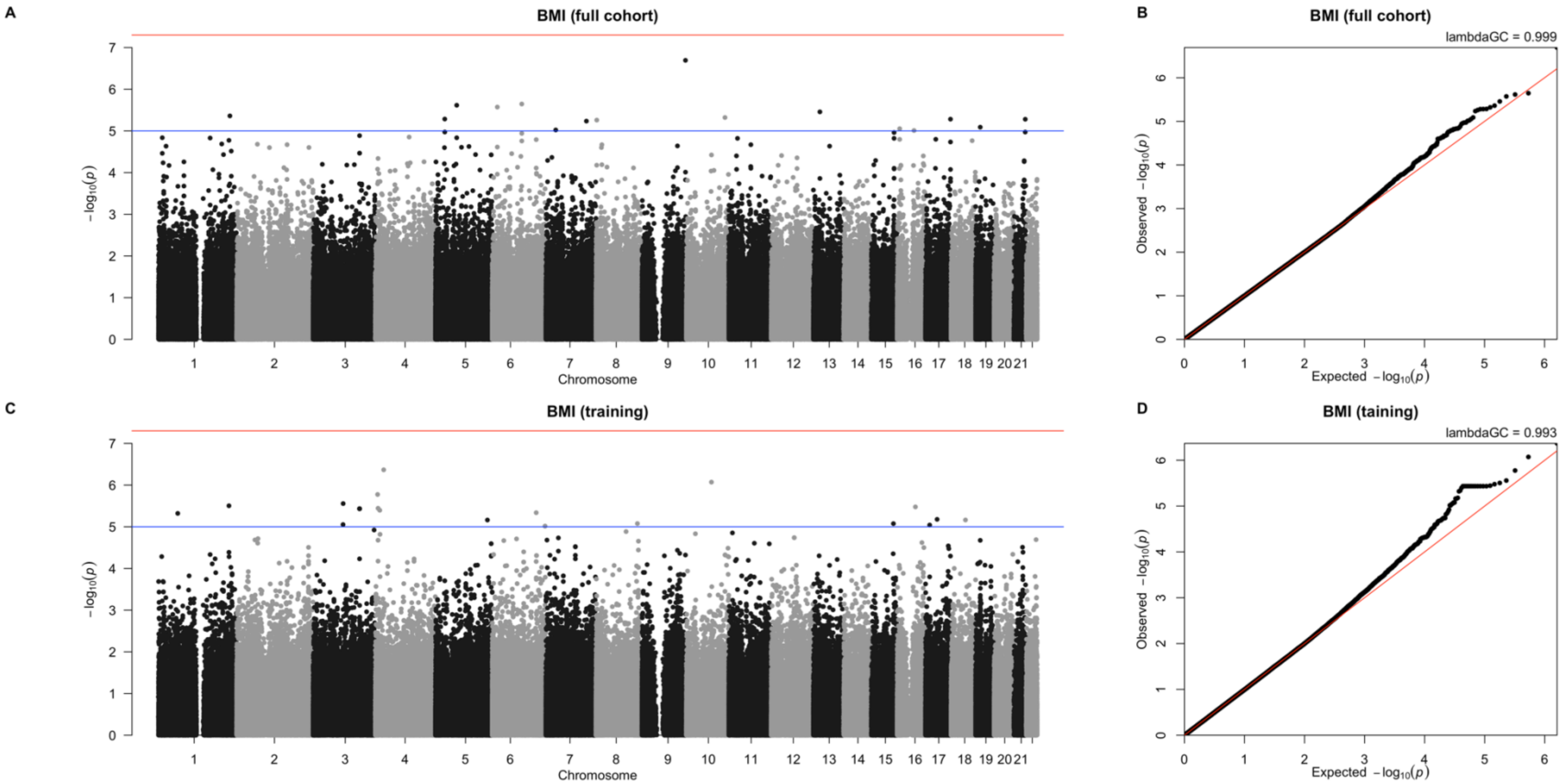

Figure S3. Genome-wide association analysis of body mass index. (A-B) Manhattan and quantile–quantile (QQ) plots of the body mass index (BMI) genome-wide association (GWAS) in the full study population. (C-D) Manhattan and QQ plots of the BMI GWAS int the training dataset. Horizontal lines in the Manhattan plots indicate the genome-wide ($p = 5 \times 10^{-8}$) and suggestive ($p = 1 \times 10^{-5}$) significance thresholds. QQ plots assessing deviation from the null distribution display observed versus expected $-\log_{10}(p)$ values, with the diagonal line representing the null expectation.
